# Supplementary material for: Improving γ-Oryzanol and γ-Aminobutyric Acid Contents in Rice Beverage Amazake Produced with Brown, Milled and Germinated Rices
Source: Foods. 2023 Mar 31;12(7):1476. doi: 10.3390/foods12071476 (PMC10094269; doi:10.3390/foods12071476)
Supplement: Supplementary file 1 [file foods-12-01476-s001.zip › foods-2184312-supplementary.pdf]

Supplementary Material (S1)

Table S1. Raw data used to fit the response surface design model according to Table 1, RSD (%), and Grubbs distance for the three repetitions. Numbers in bold represent the excluded outliers and criteria of exclusion. When the RSD criteria (>20%) was used, the selected outlier was identified through the highest Grubbs' distance (underlined values)

α-amylase activity (CU/g)

	Brown rice							Milled rice							Germinated rice						
	Raw data			RSD (%)	Grubbs' distance			Raw data			RSD (%)	Grubbs' distance			Raw data			RSD (%)	Grubbs' distance		
	R1	R2	R3		R1	R2	R3	R1	R2	R3		R1	R2	R3	R1	R2	R3		R1	R2	R3
P1	3.31	2.81	3.73	14%	0.053	1.026	0.972	**4.04**	3.05	2.39	**26%**	1.058	0.130	0.929	1.19	1.37	1.22	8%	0.748	1.136	0.388
P2	1.36	1.73	**2.14**	**23%**	0.983	0.033	1.016	1.42	1.85	1.99	17%	1.121	0.321	0.800	1.52	1.30	1.46	8%	0.822	1.113	0.291
P3	3.15	2.70	2.72	9%	1.153	0.633	0.520	2.42	2.90	2.59	9%	0.891	1.082	0.191	**1.04**	1.49	1.77	**26%**	1.068	0.153	0.915
P4	18.4	23.5	21.3	12%	1.040	0.955	0.085	18.0	19.6	23.3	13%	0.842	0.263	1.105	2.34	3.27	3.09	17%	1.136	0.748	0.388
P5	73.2	65.0	69.7	6%	0.951	1.043	0.092	72.1	71.2	94.7	17%	0.542	0.612	1.154	1.87	2.03	1.73	8%	0.032	1.016	0.983
P6	87.6	65.1	68.8	16%	1.141	0.726	0.415	88.5	74.8	79.9	9%	1.076	0.901	0.175	1.90	1.87	1.89	1%	0.887	1.084	0.196
P7	17.5	21.5	16.1	15%	0.305	1.117	0.812	18.8	**29.0**	23.0	**22%**	0.932	1.057	0.125	1.52	1.69	**0.98**	**26%**	0.330	0.793	1.123
P8	17.0	13.2	16.7	13%	0.653	1.151	0.498	17.1	13.2	19.6	19%	0.149	1.066	0.917	3.84	3.93	3.83	1%	0.525	1.153	0.628
P9	19.3	22.8	22.0	8%	1.127	0.781	0.346	18.8	20.6	**13.3**	**22%**	0.319	0.802	1.121	3.72	3.39	3.21	8%	1.080	0.187	0.893
P10	14.4	14.4	**13.6**	3%	0.591	0.563	**1.155**	3.12	3.13	3.08	1%	0.377	0.757	1.134	1.80	2.42	2.25	15%	1.117	0.812	0.305

GABA (mg/100g)

	Brown rice							Milled rice							Germinated rice						
	Raw data			RSD (%)	Grubbs' distance			Raw data			RSD (%)	Grubbs' distance			Raw data			RSD (%)	Grubbs' distance		
	R1	R2	R3		R1	R2	R3	R1	R2	R3		R1	R2	R3	R1	R2	R3		R1	R2	R3
P1	141	143	131	4%	0.394	0.743	1.137	146	175	169	9%	1.132	0.762	0.370	51	48	53	5%	0.315	1.120	0.804
P2	181	205	191	6%	0.939	1.052	0.113	185	181	178	2%	1.052	0.113	0.938	212	212	214	1%	0.450	0.696	1.146
P3	188	206	147	17%	0.256	0.847	1.103	186	155	162	10%	1.123	0.793	0.331	125	106	117	8%	0.944	1.048	0.104
P4	183	189	195	3%	0.959	0.077	1.036	169	166	163	2%	0.963	0.070	1.033	239	211	228	6%	0.928	1.059	0.132
P5	251	253	264	3%	0.725	0.416	1.141	244	205	206	10%	1.154	0.613	0.541	181	178	177	1%	1.104	0.260	0.844
P6	233	267	263	7%	1.148	0.684	0.463	224	211	215	3%	1.100	0.855	0.245	171	188	172	5%	0.631	1.153	0.522
P7	155	153	158	2%	0.020	1.010	0.990	221	206	210	4%	1.122	0.797	0.325	207	202	198	2%	1.072	0.164	0.908
P8	142	173	161	10%	1.066	0.918	0.148	**230**	198	198	9%	**1.155**	0.560	0.594	254	247	252	2%	0.787	1.125	0.338
P9	213	207	203	2%	1.055	0.122	0.934	196	199	197	1%	0.883	1.086	0.203	167	166	167	1%	0.643	1.152	0.509
P10	132	130	139	4%	0.359	0.771	1.130	195	187	196	3%	0.422	1.142	0.720	247	256	237	4%	0.038	0.980	1.018

Starch (g/100g)

	Brown rice							Milled rice							Germinated rice						
	Raw data			RSD (%)	Grubbs' distance			Raw data			RSD (%)	Grubbs' distance			Raw data			RSD (%)	Grubbs' distance		
	R1	R2	R3		R1	R2	R3	R1	R2	R3		R1	R2	R3	R1	R2	R3		R1	R2	R3
P1	65.9	66.0	66.8	1%	0.718	0.425	1.142	65.3	64.5	66.5	2%	0.130	0.929	1.059	73.8	70.3	71.6	2%	1.073	0.906	0.167
P2	67.5	67.3	67.5	0%	0.327	1.123	0.795	**61.8**	62.2	62.2	0%	**1.155**	0.582	0.573	71.4	72.3	72.5	1%	1.146	0.447	0.698
P3	62.0	63.6	62.7	1%	0.988	1.011	0.023	65.8	65.5	65.0	1%	0.963	0.071	1.033	72.8	64.7	65.2	7%	1.154	0.622	0.531
P4	59.0	58.8	59.0	0%	0.639	1.152	0.514	58.4	58.9	58.2	1%	0.349	1.128	0.779	64.8	63.2	63.4	1%	1.145	0.703	0.442
P5	56.0	55.9	56.0	0%	0.456	1.147	0.691	55.9	55.4	56.0	1%	0.377	1.134	0.757	63.5	62.9	61.7	2%	0.866	0.228	1.094
P6	56.0	55.8	56.0	0%	0.670	1.149	0.480	56.1	55.5	55.5	1%	1.146	0.452	0.694	61.6	63.5	62.9	2%	1.097	0.862	0.235
P7	65.5	**65.0**	65.5	0%	0.581	**1.155**	0.574	58.6	59.2	58.9	1%	0.925	1.061	0.136	61.0	61.6	58.9	2%	0.370	0.762	1.132
P8	52.5	52.6	53.0	0%	0.622	0.531	1.154	63.4	61.9	63.4	1%	0.526	1.153	0.627	58.7	58.7	**61.2**	2%	0.588	0.566	**1.155**
P9	59.3	59.0	58.7	0%	0.980	0.038	1.019	67.3	66.7	67.5	1%	0.386	1.135	0.750	61.6	61.6	62.0	0%	0.507	0.654	1.152
P10	65.8	66.0	66.2	0%	1.067	0.152	0.915	59.6	59.5	58.8	1%	0.660	0.491	1.151	58.0	58.1	58.3	%	0.948	0.097	1.045
